# Long-term outcomes of cardiogenic shock and cardiac arrest complicating ST-elevation myocardial infarction according to timing of occurrence

**DOI:** 10.1093/ehjopen/oeae075

**Published:** 2024-09-03

**Authors:** Gabriel Kanhouche, Jose Carlos Nicolau, Remo Holanda de Mendonça Furtado, Luiz Sérgio Carvalho, Talia Falcão Dalçoquio, Brunna Pileggi, Mauricio Felippi de Sa Marchi, Pedro Abi-Kair, Neuza Lopes, Roberto Rocha Giraldez, Luciano Moreira Baracioli, Felipe Gallego Lima, Ludhmila Abrahão Hajjar, Roberto Kalil Filho, Fábio Sandoli de Brito Junior, Alexandre Abizaid, Henrique Barbosa Ribeiro

**Affiliations:** Department of Interventional Cardiology, Heart Institute (InCor), Hospital das Clinicas HCFMUSP, Faculdade de Medicina, Universidade de Sao Paulo, Sao Paulo, SP, Brazil; Department of Interventional Cardiology, Heart Institute (InCor), Hospital das Clinicas HCFMUSP, Faculdade de Medicina, Universidade de Sao Paulo, Sao Paulo, SP, Brazil; Department of Interventional Cardiology, Heart Institute (InCor), Hospital das Clinicas HCFMUSP, Faculdade de Medicina, Universidade de Sao Paulo, Sao Paulo, SP, Brazil; Department of Cardiology, Brazilian Clinical Research Institute, São Paulo, Brazil; Department of Interventional Cardiology, Heart Institute (InCor), Hospital das Clinicas HCFMUSP, Faculdade de Medicina, Universidade de Sao Paulo, Sao Paulo, SP, Brazil; Department of Interventional Cardiology, Heart Institute (InCor), Hospital das Clinicas HCFMUSP, Faculdade de Medicina, Universidade de Sao Paulo, Sao Paulo, SP, Brazil; Department of Interventional Cardiology, Heart Institute (InCor), Hospital das Clinicas HCFMUSP, Faculdade de Medicina, Universidade de Sao Paulo, Sao Paulo, SP, Brazil; Department of Interventional Cardiology, Heart Institute (InCor), Hospital das Clinicas HCFMUSP, Faculdade de Medicina, Universidade de Sao Paulo, Sao Paulo, SP, Brazil; Department of Interventional Cardiology, Heart Institute (InCor), Hospital das Clinicas HCFMUSP, Faculdade de Medicina, Universidade de Sao Paulo, Sao Paulo, SP, Brazil; Department of Interventional Cardiology, Heart Institute (InCor), Hospital das Clinicas HCFMUSP, Faculdade de Medicina, Universidade de Sao Paulo, Sao Paulo, SP, Brazil; Department of Interventional Cardiology, Heart Institute (InCor), Hospital das Clinicas HCFMUSP, Faculdade de Medicina, Universidade de Sao Paulo, Sao Paulo, SP, Brazil; Department of Interventional Cardiology, Heart Institute (InCor), Hospital das Clinicas HCFMUSP, Faculdade de Medicina, Universidade de Sao Paulo, Sao Paulo, SP, Brazil; Department of Interventional Cardiology, Heart Institute (InCor), Hospital das Clinicas HCFMUSP, Faculdade de Medicina, Universidade de Sao Paulo, Sao Paulo, SP, Brazil; Department of Interventional Cardiology, Heart Institute (InCor), Hospital das Clinicas HCFMUSP, Faculdade de Medicina, Universidade de Sao Paulo, Sao Paulo, SP, Brazil; Department of Interventional Cardiology, Heart Institute (InCor), Hospital das Clinicas HCFMUSP, Faculdade de Medicina, Universidade de Sao Paulo, Sao Paulo, SP, Brazil; Department of Interventional Cardiology, Heart Institute (InCor), Hospital das Clinicas HCFMUSP, Faculdade de Medicina, Universidade de Sao Paulo, Sao Paulo, SP, Brazil; Department of Interventional Cardiology, Heart Institute (InCor), Hospital das Clinicas HCFMUSP, Faculdade de Medicina, Universidade de Sao Paulo, Sao Paulo, SP, Brazil; Department of Interventional Cardiology, Heart Institute (InCor), Hospital das Clinicas HCFMUSP, Faculdade de Medicina, Universidade de Sao Paulo, Sao Paulo, SP, Brazil

**Keywords:** STEMI cardiogenic shock, STEMI cardiac arrest, Myocardial infarction, Acute coronary syndrome, STEMI long-term survival

## Abstract

**Aims:**

Cardiogenic shock (CS) and cardiac arrest (CA) are serious complications in ST-elevation myocardial infarction (STEMI) patients, with lack of long-term data according to their timing of occurrence. This study sought to determine the incidence and relationship between the timing of occurrence and prognostic impact of CS and CA complicating STEMI in the long-term follow-up.

**Methods and results:**

We conducted a retrospective analysis of consecutive STEMI patients treated between 2004 and 2017. Patients were divided into four groups based on the occurrence of neither CA nor CS, CA only, CS only, and both CA and CS (CA−CS−, CA+, CS+, and CA+CS+, respectively). Adjusted Cox regression analysis was used to assess the independent association between the CS and CA categories and mortality. A total of 1603 STEMI patients were followed for a median of 3.6 years. CA and CS occurred in the 12.2% and 15.9% of patients, and both impacted long-term mortality [adjusted hazard ratio (HR) = 2.59, 95% confidence interval (CI): 1.53–4.41, *P* < 0.001; HR = 3.16, 95% CI: 2.21–4.53, *P* < 0.001, respectively). CA+CS+ occurred in 7.3%, with the strongest association with higher mortality (adjusted HR = 5.36; 95% CI: 3.80–7.55, *P* < 0.001). Using flexible parametric models with B-splines, the increased mortality was restricted to the first ∼10 months. In addition, overall mortality rates were higher at all timings (all with *P* < 0.001), except for CA during initial cardiac catheterization (*P* < 0.183).

**Conclusion:**

CS and CA complicating patients presenting with STEMI were associated with higher long-term mortality rate, especially in the first 10 months. Both CS+ and CA+ at any timeframe impacted outcomes, except for CA+ during the initial cardiac catheterization, although this will have to be confirmed in larger future studies, given the relatively small number of patients.

## Graphical abstract

**Graphical Abstract:**
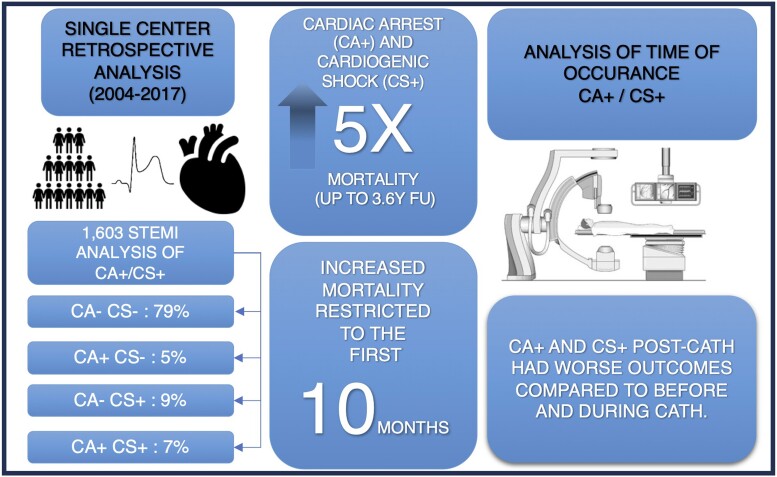
Population and proportion of cardiogenic shock and cardiac arrest in ST-elevation myocardial infarction patients. Cardiogenic shock and cardiac arrest are the group with worse outcome. Both complications have higher mortality when occurring after initial cardiac catheterization.

## Introduction

ST-elevation myocardial infarction (STEMI) in the spectrum of patients with acute coronary syndrome (ACS) represents the subtype with the worst short-term clinical outcomes.^1,2^ In the last decades, there has been an increase in devices and reperfusion therapies, together with guideline-oriented medical treatment, which substantially diminished short-term mortality and readmissions in such patients.^3,4^ Yet, mortality rates remain exceptionally high for STEMI patients who develop cardiogenic shock (CS) and/or cardiac arrest (CA).^5^

CS is characterized by the heart’s inability to maintain adequate blood supply for the body’s metabolic demands, and STEMI is the most frequent cause of this complication (∼10% of STEMI patients). It can develop at any time during the STEMI course, with in-hospital and 1-year mortality rates that can reach up to 35–45% and 55–60% of patients, respectively. Despite treatment advances in recent decades, a minimal improvement in mortality has been achieved in CS patients.^6–8^

CA is another common STEMI complication with also high mortality rates and which may result in severe neurological damage, particularly in cases of non-shockable rhythm.^9^ Approximately 5–8% STEMI patients suffer CA, of which up to 20–40% will eventually die.^9,10^ The most common pathogenesis in CA is coronary atherosclerosis in up to 70% of events.^11^ Likewise, CA and CS may also present at different time frames in STEMI patients (pre-cardiac catheterization, during- and after the procedure), which represent different challenges for maintaining tissue perfusion and therefore for the management of this group of patients.

Although CS and CA short-term outcomes are well described in the literature, little is known about their impact on long-term outcomes. Most cohorts are small and rarely present data beyond 1-year follow-up.^12^ Importantly, CS and CA are often excluded in observational registries, so that data on their frequency and timing of occurrence is lacking, in addition to their impact on the long-term clinical outcomes.^13^ Therefore, this study sought to determine the incidence, timing of occurrence, and prognostic impact of in-hospital CS and CA complicating STEMI in the long-term follow-up.

## Methods

### Study population and protocol

**Figure 1:**
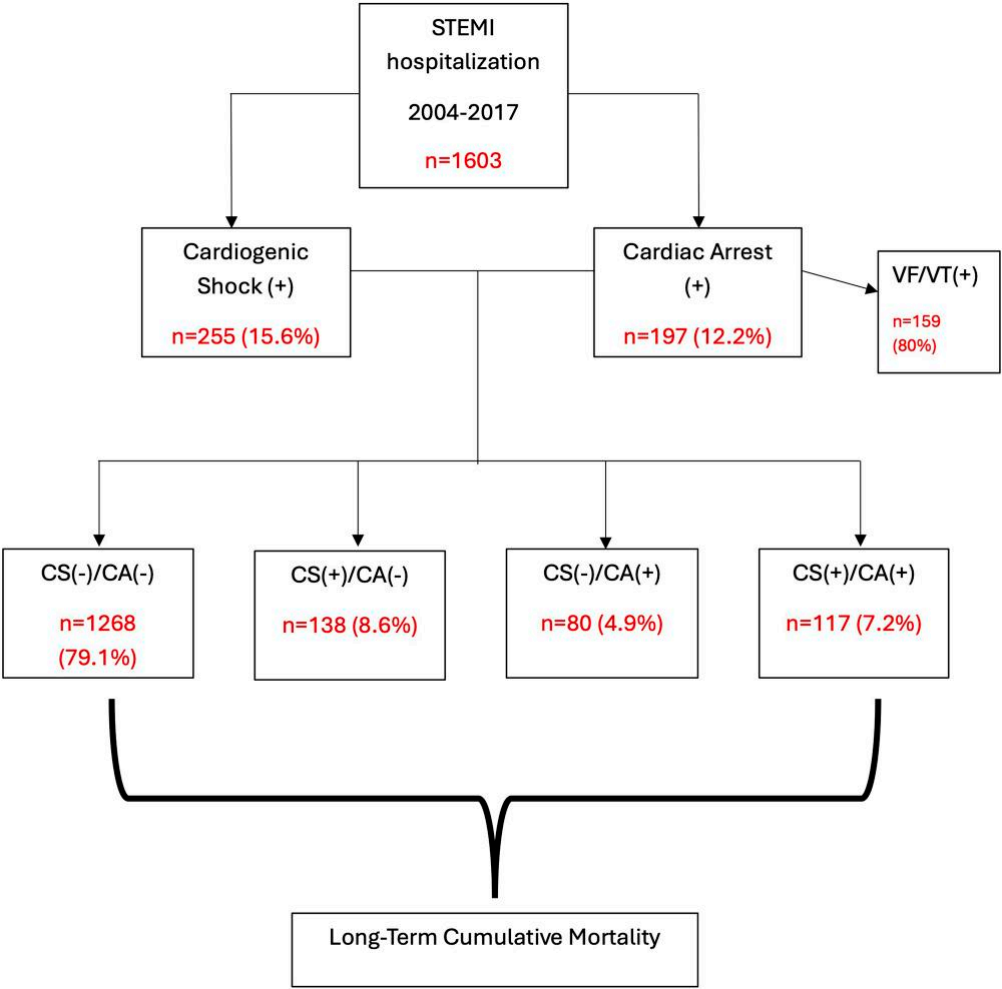
Flowchart of the study population.

From January 2004 to December 2017, a total of 1603 consecutive all-comer STEMI patients were included into our database. *Figure 1* shows the flow diagram of the study population. Patients were divided into four groups based on the occurrence of neither CA nor CS, CS only, CA only, and both CS and CA (CA−CS−, CS+, CA+, and CS+CA+, respectively). We also evaluated the patients according to the timing of occurrence of CS and/or CA with respect to the initial cardiac catheterization as pre-, during, or post-procedure. CS/CA pre-catheterization included the events occurring either at the referral centre, during transfer, or at our hospital admission, so that out-of-hospital events were excluded. Clinical follow-up was carried out by clinical visits and/or through phone contact at 1-month, 6- to 12-month post-STEMI, and yearly thereafter. The primary outcome was the long-term mortality after hospital discharge, and in-hospital mortality rates were also evaluated.

### Definitions

STEMI diagnosis followed initially the classical definition of the World Heart Federation^14^ and, from 2008 on, the (first to fourth) Universal Definition of Myocardial Infarction.^15^ CS was defined as a systolic blood pressure < 90 mmHg for 1 h not fluid responsive, thought to be secondary to cardiac dysfunction, and associated with signs of hypoperfusion or cardiac index < 2.2 L/min/mm^2^ and pulmonary capillary wedge pressure > 18 mmHg or overt pulmonary congestion at physical examination or chest imaging.^16^ It includes patients in Group C or above according to the Society for Cardiovascular, Angiography & Interventions (SCAI) shock classification.^17^

CA was defined as cessation of cardiac activity with no signs of circulation. It is divided in shockable or non-shockable rhythm.^18^ When primary percutaneous coronary intervention (PPCI) was not achievable, the decision of whether non-PPCI, medical treatment, or coronary artery bypass graft (CABG) was performed was left to the discretion of the heart team according to each patients’ clinical and anatomical characteristics. Patients were submitted to mechanical support with intra-aortic balloon pump (IABP) at the operator's clinical discretion. No patients underwent Impella or extracorporeal membrane oxygenation (ECMO).

### Statistical analysis

STEMI patients were classified according to the presence (+) or absence (−) of CS and/or CA. We used multiple imputations (mice package in R) to handle missing values. A predictive mean matching model was used for numeric variables, logistic regression for binary variables (with two levels), and Bayesian polytomous regression for factor variables (≥2 levels). Imputed values, residual distribution, and convergence coefficients were checked. We did not impute missing values for the outcomes. The imputation step resulted in five complete data sets, each of which contains different estimates of the missing values for all 1603 patients. After imputation, we pooled and merged all five data sets to perform stepwise Cox regressions. Normally distributed data are presented as mean and standard deviation and skewed data as median and 25th–75th percentiles. Distribution normality and variance were checked using histograms, Kolmogorov–Smirnoff test, normal probability plots, and residual scatter plots. *χ*^2^, Kruskal–Wallis, or two-tailed analysis of variance were used for comparison of baseline data, respectively, for categoric data, continuous and non-normally distributed variables, and continuous and normally distributed variables, respectively.

Survival rates are presented as Kaplan–Meier plots, and differences between groups were tested by the log-rank test. We also performed Cox regression models to identify the independent mortality impact of CA and CS timing. Short-term and long-term mortality were assessed using landmark analyses. We used B-splines to define the best cut-off point for a landmark that represents the period when the hazard remains stable (stable variance). After defining the best cut-off, we used landmark analyses in four different scenarios: (i) from hospital admission (time zero) to discharge (in-hospital mortality), (ii) from time of hospital discharge (time zero) to death or censoring (post-discharge mortality), (iii) from hospital admission (time zero) to 9.9 months, and (iv) from 9.9 months to death or censoring. Finally, we did a stepwise Cox regression model to identify key predictors of all-cause mortality. Complete follow-up rates were 96.4% at 30 days and 81.2% at 4 years. All tests are two-tailed and *P*-values <0.05 were considered as statistically significant. Analyses were carried out using R (v3.5.3) language and R Studio v1.1.4.

### Compliance with ethical standards

This study conformed to the International Council on Harmonization norms on medical research in humans. The study was approved by the Ethics Committee from Clinics Hospital. Because the data are based on individual anonymized information obtained for administrative purposes, informed consent was waived, according to local regulations.

## Results

### Baseline clinical and angiographic characteristics

Baseline clinical characteristics of the study population are shown in *Table 1*. A total of 255 (15.6%) patients presented CS+ and 197 (12.2%) presented CA+, of whom 138 (8.6%) had CS+ only, 80 (4.9%) presented CA+ only, and 117 (7.2%) presented both CS+CA+. The majority of CS and CA occurred pre-cardiac catheterization. Most patients were male, with a mean age of ∼60 years, although slightly younger (*P* < 0.001) with more females (*P* = 0.004) in the CS+ group as compared with CA+. Furthermore, CS+ and CS+CA+ patients presented higher prevalence of diabetes and prior history of heart failure, and less dyslipidaemia as compared with CA+ and CA−CS− patients (all with *P* < 0.05). Also, no significant differences were seen with respect to medication use at baseline.

**Table 1:** Baseline characteristics of the study population

	No cardiac arrest or shock	Cardiac arrest+	Cardiogenic shock+	Cardiac arrest+ and cardiogenic shock+	*P*
*N*	1263 (79.1%)	80 (4.9%)	138 (8.6%)	117 (7.2%)	
**Demography**					
Age (years)	60.45 ± 12.54^a,b^	58.36 ± 12.48	64.47 ± 13.53^a^	63.56 ± 12.10^b^	**<0.001**
Male sex	925 (73.2)^a^	60 (75.0)	81 (58.7)^a^	85 (72.6)	**0.004**
**Past medical history**					
ACS	243 (19.2)	15 (18.8)	37 (26.8)	23 (19.7)	0.208
PCI	168 (13.3)	9 (11.2)	24 (17.4)	12 (10.3)	0.358
CABG	85 (6.7)	3 (3.8)	8 (5.8)	5 (4.3)	0.543
Heart failure	69 (5.5) ^a^	3 (3.8)	16 (11.6) ^a^	8 (6.8)	**0.027**
Diabetes mellitus	349 (27.6) ^a,b^	18 (22.5)	45 (32.6)	45 (38.5) ^b^	**0.034**
Insulin dependent	60 (4.8)	3 (3.8)	5 (3.6)	5 (4.3)	0.913
CKD	59 (6.9)	8 (10.0)	17 (13.8)	6 (11.3)	0.068
Stroke	66 (5.2)	6 (7.5)	12 (8.7)	8 (6.8)	0.314
Hypertension	843 (66.7)	59 (73.8)	95 (68.8)	82 (70.1)	0.528
Dyslipidaemia	610 (48.3)^b^	36 (45.0)	61 (44.2)	41 (35.0)^b^	**0.044**
Smoking	411 (32.5)	29 (36.2)	41 (29.7)	42 (35.9)	0.665
Chronic obstructive pulmonary disease	45 (3.6)	3 (3.8)	5 (3.6)	8 (6.8)	0.096
PVD	40 (3.1)	3 (3.8)	5 (3.6)	6 (5.1)	0.164
**Medications prior to admission**					
Statin	245 (29.4)	22 (30.0)	24 (28.6)	11 (20.8)	0.432
Clopidogrel	40 (4.8)	5 (6.3)	5 (6.0)	3 (5.7)	0.461
Aspirin	223 (26.7)	25 (31.3)	23 (27.4)	10 (18.9)	0.668
Beta-blocker	214 (25.7)	21 (26.3)	21 (25.0)	11 (20.8)	0.501

Values are *n* (%), mean ± SD.

ACS, acute coronary syndrome; CABG, coronary artery bypass graft; CKD, chronic kidney disease; PCI, percutaneous coronary intervention; PVD, peripheral vascular disease.

^a^Bonferroni *post hoc* analysis if *P* < 0.05 for ‘no cardiac arrest or shock’ vs. ‘cardiogenic shock+’.

^b^Bonferroni *post hoc* analysis if *P* < 0.05 for ‘no cardiac arrest or shock’ vs. ‘cardiac arrest+ and cardiogenic shock+’.

Baseline STEMI event characteristics are shown in *Table 2*. Most STEMI involved the anterior wall and the left anterior descending artery, with similar rates among the groups, followed by the right coronary artery. Furthermore, all groups except CA+ had more frequently multi-vessel coronary artery disease as the most common CAD pattern, while CA+ more likely had one vessel disease. Moreover, CS+ and CS+CA+ patients presented in worse clinical conditions, yielding higher baseline thrombolysis in myocardial infarction (TIMI) risk score (3.8 ± 2.1, 4.0 ± 2.1, 6.5 ± 2.7, and 6.7 ± 2.8, respectively; *P* < 0.001), higher mean heart rates, lower mean systolic blood pressure, and diastolic blood pressure (all with *P* < 0.001).

**Table 2:** Baseline ST-elevation myocardial infarction event characteristics

	No cardiac arrest or shock	Cardiac arrest+	Cardiogenic shock+	Cardiac arrest+ and cardiogenic shock+	*P*
*N*	1263 (79.1%)	80 (4.9%)	138 (8.6%)	117 (7.2%)	
Anterior wall STEMI	602 (47.7)	38 (47.5)	82 (59.4)	61 (52.1)	0.059
TIMI risk	3.75 ± 2.13^c,d^	4.03 ± 2.13	6.49 ± 2.74^c^	6.65 ± 2.77^d^	**<0.001**
SBP (mmHg)	132.0 ± 28.5^c,d^	126.0 ± 26.3	112.2 ± 26.5^c^	108.6 ± 26.8^d^	**<0.001**
DBP (mmHg)	79.9 ± 17.3^c,d^	77.9 ± 15.2	71.2 ± 16.3^c^	69.3 ± 19.1^d^	**<0.001**
HR (b.p.m.)	78.9 ± 18.3^c,d^	80.8 ± 15.7	89.2 ± 23.5^c^	88.0 ± 23.5^d^	**<0.001**
Time pain-first hospital admission (min)	150 [60 420]^b^	113 [43 356]^b^	180 [90 480]	120 [52420]	**0.008**
Time pain-PPCI (min)	373 [220 780]^b,c^	297 [128 420]^b^	547 [241 863]^c^	360 [241 555]	**0.013**
Time door-PPCI (min)^a^	87 [54–151]	80 [45–139]	93 [60–182]	78 [56–165]	0.476
**Type of treatment**					
PCI	1012 (80.1)^b,c^	70 (87.5)^b^	99 (71.7)^c^	99 (84.6)	**0.016**
CABG	123 (9.7)^b,c^	2 (2.5)^b^	21 (15.2)^c^	10 (8.5)	**0.022**
Medical Rx	128 (10.1)	8 (10.0)	18 (13.0)	8 (6.8)	0.444
**Number of diseased vessels**					
None	137 (10.8)	6 (7.5)	16 (11.6)	16 (13.7)	0.585
One vessel	359 (28.4)^b,c^	33 (41.2)^b^	28 (20.3)^c^	26 (22.2)	**0.004**
Two vessels	311 (24.6)	14 (17.5)	36 (26.1)	28 (23.9)	0.507
Multi-vessel	456 (36.1)	27 (33.8)	58 (42.0)	47 (40.2)	0.427
**Target territory**					
LAD	516 (40.9)	34 (42.5)	59 (42.8)	53 (45.3)	0.794
LCX	122 (9.7)	5 (6.2)	9 (6.5)	7 (6.0)	0.289
RCA	340 (26.9)	29 (36.2)	30 (21.7)	34 (29.1)	0.130
LMC	11 (0.9)	0 (0.0)	2 (1.4)	2 (1.7)	0.582
Graft	29 (2.3)	0 (0.0)	2 (1.4)	3 (2.6)	0.508
LIMA	6 (0.5)	0 (0.0)	0 (0.0)	0 (0.0)	0.660
**End-procedure TIMI flow**					**0.041**
0	21 (2.0)	5 (7.1)	0 (0)	4 (4.0)	
1	7 (0.7)	1 (1.4)	0 (0)	2 (2.0)	
2	17 (1.7)	5 (7.1)	0 (0)	2 (2.0)	
3	921 (91.0)^d^	66 (94.2)	92 (91.9)	72 (72)^d^	

Values are *n* (%), mean ± SD or median [IQR].

CABG, coronary artery bypass graft; DBP, diastolic blood pressure; HR, heart rate; LCX, left circumflex; LDA, left descending artery; LIMA, left internal mammary artery; LMC, left main coronary; PCI, percutaneous coronary intervention; RCA, right coronary artery; SBP, systolic blood pressure; STEMI, ST-elevation myocardial infarction; TIMI risk, thrombolysis in myocardial infarction.

^a^This time refers to the admission to our hospital where PPCI was performed.

^b^Bonferroni *post hoc* analysis if *P* < 0.05 for ‘no cardiac arrest or shock’ vs. ‘cardiac arrest+’.

^c^Bonferroni *post hoc* analysis if *P* < 0.05 for ‘no cardiac arrest or shock’ vs. ‘cardiogenic shock+’.

^d^Bonferroni *post hoc* analysis if *P* < 0.05 for ‘no cardiac arrest or shock’ vs. ‘cardiac arrest+ and cardiogenic shock+’.

The most prevalent treatment option in all groups was PCI in ∼80% of the patients, being primary PCI in 49.6%, followed by CABG in ∼10%. PCI was more often indicated in the CA+ group (87.2%; *P* = 0.016), while CABG was more frequent in the CS+ groups (15.2%; *P* = 0.022). Among patients undergoing PCI, the final TIMI 3 flow was less frequently observed in the CS+CA+ group vs. CS+ and CA+ (72.0% vs. 91.9% vs. 94.2%, respectively; *P* = 0.041). Patients undergoing thrombolysis (35.8%) were referred to our hospital for pharmaco-invasive strategies.

### Clinical outcomes

#### In-hospital outcomes

As expected, CA+, CS+, and CS+CA+ patients had worse in-hospital outcomes compared with CA−CS− patients (3 deaths, 16 deaths, 24 deaths, and 8 deaths, respectively). Including all in-hospital deaths in STEMI patients, 86% were related to CS and/or CA, and almost half of this was related to both CS+CA+. Therefore, most deaths in the CS+ and CA+ groups occurred in-hospital (see Supplementary material online, *Figure S1*). As shown in *Table 3*, these three groups had longer hospitalization length, including critical care unit stay. They also had greater incidence of sepsis, acute renal injury, and need for renal replacement therapy, especially among patients with CS (CS+: 9.2%; CS+CA+: 20.0%; *P* < 0.001). Furthermore, these patients presented more major bleeding events, requiring blood transfusion, particularly in the CS+CA+ group with 13.2% and 17.5% rates, respectively (*P* < 0.001).

**Table 3:** In-hospital outcomes

	No cardiac arrest or shock	Cardiac arrest+	Cardiogenic shock+	Cardiac arrest+ and cardiogenic shock+	*P*
*N*	1263 (79.1%)	80 (4.9%)	138 (8.6%)	117 (7.2%)	
Hospitalization (days)	10.2 ± 10.4^c,d^	14.6 ± 16.9	25.8 ± 24.9^c^	23.7 ± 23.5^d^	**<0.001**
CCU (days)	6.8 ± 7.0^b,c,d^	11.85 ± 15.1^b^	20.9 ± 21.6^c^	18.4 ± 17.8^d^	**<0.001**
LVEF (%)	47 ± 11^c,d^	48 ± 11	38 ± 12^c^	39 ± 12^d^	**<0.001**
IABP	18 (1.5)^c,d^	4 (5.1)	72 (53.7)^c^	68 (59.6)^d^	**<0.001**
In-hospital AMI recurrence	114 (9.0)	7 (8.8)	10 (7.2)	3 (2.6)	0.108
Mechanical complications^a^	35 (2.8)	0 (0.0)	8 (5.8)	6 (5.1)	0.001
Mechanical ventilation	31(2.5)^b,c,d^	15(18.8)^b^	55(39.9)^c^	83(70.9)^d^	**<0.001**
Stroke	10 (0.9)	0 (0.0)	3 (2.2)	2 (1.8)	0.289
Complete heart block	33 (2.8)^d^	7 (9.0)	13 (9.7)	19 (16.5)^d^	**<0.001**
Temporary pacemaker	50 (4.0)^b,c,d^	12 (15.0)^b^	20 (14.5)^c^	29 (25.2)^d^	**<0.001**
Permanent pacemaker	1 (2.0)	1 (9.1)	1 (5.0)	1 (3.6)	0.694
Atrial fibrillation/flutter	55 (4.7)^b,c,d^	9 (11.5)^b^	28 (20.9)^c^	15 (13.2)^d^	**<0.001**
Sepsis	49 (4.2)^c,d^	10 (12.8)	55 (41.0)^c^	49 (43.0)^d^	**<0.001**
Acute renal failure	69 (5.9)^c,d^	9 (11.5)	48 (35.8)^c^	50 (43.9)^d^	**<0.001**
Dialysis	14 (1.3)^d^	2 (2.8)	11 (9.2)	20 (20.0)^d^	**<0.001**
Major bleeding	18 (1.5)^d^	5 (6.4)	9 (6.7)	15 (13.2)^d^	**<0.001**
Blood transfusion	14 (1.1)^c,d^	2 (4.8)	9 (10.5)^c^	11 (17.5)^d^	**<0.001**
Overall death	127 (10.1)^b,c,d^	16 (20.0)^b^	51 (37.0)^c^	61 (52.1)^d^	**<0.001**
Cardiovascular death	21 (1.7)^c,d^	4 (5.0)	29 (21.0)^c^	43 (36.8)^d^	**<0.001**
Death due bleeding	2 (0.2)	1 (1.2)	0 (0.0)	1 (0.9)	0.128
Death due sepsis	3 (0.2)^c,d^	3 (3.8)	15 (10.9)^c^	20 (17.1)^d^	**<0.001**

Values are *n* (%), mean ± SD or median [IQR].

AMI, acute myocardial infarction; CCU critical care unit; CV death, cardiovascular death; IABP, intra-aortic balloon pump; LVEF, left ventricular ejection fraction; sustained VT, sustained ventricular tachycardia.

^a^Mechanical complications included: cardiac tamponade, intraventricular communication, cardiac perforation, and mitral regurgitation.

^b^Bonferroni *post hoc* analysis if *P* < 0.05 for ‘no cardiac arrest or shock’ vs. ‘cardiac arrest+’.

^c^Bonferroni *post hoc* analysis if *P* < 0.05 for ‘no cardiac arrest or shock’ vs. ‘cardiogenic shock+’.

^d^Bonferroni *post hoc* analysis if *P* < 0.05 for ‘no cardiac arrest or shock’ vs. ‘cardiac arrest+ and cardiogenic shock+’.

Among all groups, those with CS+ and CS+CA+ had lower left ventricular ejection fraction and IABP was implanted in more than half of the patients (CS+: 53.7%; CS+CA+: 59.6%; *P* < 0.001). Despite the more frequent use of mechanical circulatory support, these patients had greater mortality rates (CS+: 37.0%; CA+CS+: 52.1%, respectively). Including all in-hospital deaths in STEMI patients, 86% were related to CS and/or CA, and almost half of this was related to both CS+CA+. Medication use at hospital discharge is demonstrated in Supplementary material online, *Table S1*.

We also compared results across two time periods (2004–09 and 2010–17). As time progressed, there were less hospital stay, fewer mechanical complications, and lower overall and cardiac mortality. (see Supplementary material online, *Table S2*).

### Predictors of long-term mortality and the impact of CS+ and CA+

**Figure 2:**
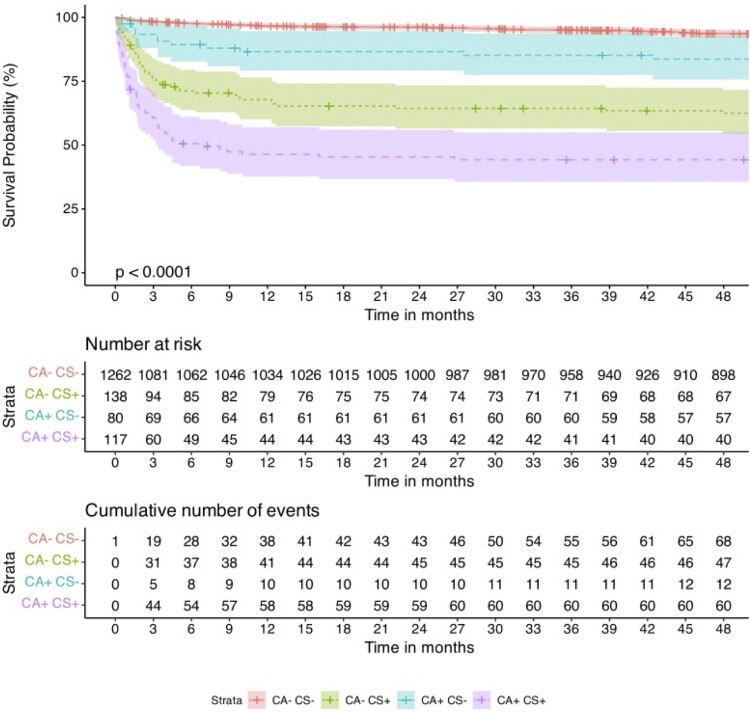
Kaplan–Meier survival at the long-term follow-up according to the presence of cardiogenic shock (CS+), cardiac arrest (CA), or both (CA+CS+).

**Figure 3:**
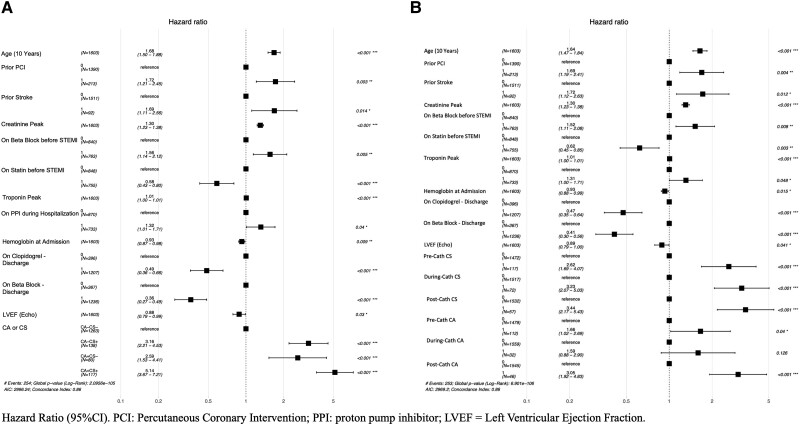
Stepwise Cox regression model for all-cause long-term mortality. (*A*) Multivariate analysis including the strongest independent risk factors for mortality during a 48-month follow-up (concordance index of 0.86). (*B*) Multivariate analysis including CS+ and/or CA+ time of occurrence as an independent risk factor for long-term mortality (concordance index of 0.86).

Kaplan–Meier survival curves are shown in *Figure 2*, according to the presence or absence of CS+, CA+, or CS+CA+. At 4-year follow-up, the occurrence of CS+, CA+, and CS+CA+ were associated with lower survival rate (log-rank: *P* < 0.0001). Over a median follow-up of 3.6 years, stepwise Cox regression model for all-cause mortality, adjusted for various covariates, confirmed that CS+ [hazard ratio (HR): 3.16; 95% confidence interval (CI): 2.21–4.53, *P* < 0.001] and CA+ (HR: 2.59; 95% CI: 1.53–4.41, *P* < 0.001) were significantly associated with higher mortality rates among STEMI patients (*Figure 3A*; Supplementary material online, *Figures S2* and *S4*). When combined CS+ and CA+, the risk of death increased five-fold (HR: 5.14; 95% CI: 3.67–7.21, *P* < 0.001) (*Graphical Abstract*). In addition, age, prior PCI, prior stroke, peak creatinine, beta-blocker use before STEMI, proton pump inhibition use during hospitalization, and anaemia at admission were significant independently factors associated with greater mortality (all with *P* < 0.05). On the contrary, statin use before STEMI and the use of p2y12 inhibitor and beta-blocker at discharge were protective factors (all with *P* < 0.001).

### Landmark analyses and time-varying hazard ratio analyses

**Figure 4:**
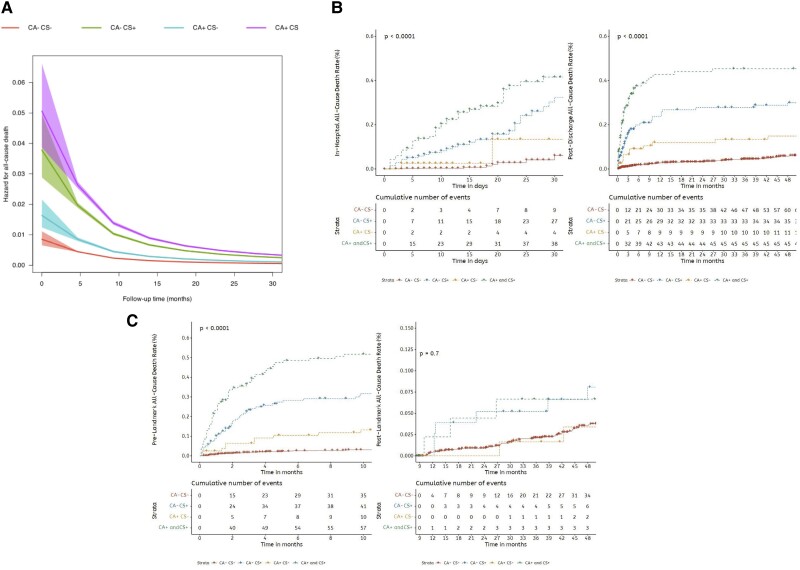
Flexible parametric model with B-splines (*A*) and landmark analyses at discharge (*B*) and after 10 months (*C*) for cumulative mortality at the long-term follow-up, comparing the four groups (CA−CS−, CA+CS−, CA−CS+, or CA+CS+).

Using a flexible parametric model with B-splines, we estimated time-varying hazard for all-cause death, and this revealed that CS+ and CA+ were associated with higher mortality in the initial 9.9 months after the index event (*Figure 4A*; *Graphical Abstract*). In Kaplan–Meier, mortality curves considering starting follow-up at hospital discharge, CS+, CA+, and CS+CA+ were still significantly associated with higher mortality rates (*Figure 4B*). Nonetheless, after excluding the initial 10 months, CS+, CA+, and CS+CA+ were no longer associated with higher mortality (log-rank: *P* = 0.7) (*Figure 4C*).

### Impact of time of occurrence of CS/CA in the cumulative mortality

The timing of occurrence of both CS+ and CA+ is shown in *Graphical Abstract* and Supplementary material online, *Figure S3*. Majority of CS+ and CA+ occurred pre-catheterization (38.5% and 59.0% of patients, respectively; *P* < 0.001). Stepwise Cox regression model for all-cause long-term mortality according to the impact of CS and CA timing of occurrence is presented in *Figure 3B*. CS+ was associated with higher overall mortality rate irrespective of occurrence timing, although CS post-cardiac catheterization had even worse clinical outcomes (HR: 3.55; 95% CI: 2.24–5.63, *P* < 0.001). On the contrary, CA+ during the procedure was not associated with overall mortality (HR: 1.59; 95% CI: 0.88–2.90, *P* < 0.126). On the other hand, both CA pre-cardiac catheterization and post-cardiac catheterization were associated with higher overall mortality during the follow-up (*Figure 3B*).

## Discussion

The main findings of the present study were as follows: (i) CS and CA among STEMI patients occurred in ∼20% of patients, especially before cardiac catheterization, with a substantial impact in mortality; (ii) isolated CS+ or CA+ was associated with approximately three-fold higher mortality rates, while the combination of CA+CS+ was associated with five-fold greater mortality in the long-term follow-up (*Figure 3A*); (iii) 60% of the deaths occurred in the in-hospital phase, although a residual impact in mortality was seen up to ∼10 months after STEMI among patients presenting CS+ and/or CA+; (iv) with respect to the CS and CA timing of occurrence, CS impacted mortality in all time frames, although CA only before and after cardiac catheterization impacted mortality, but not during the procedure.

Despite advances in the last decades for the treatment of ACS, CS and CA complicating STEMI patients are still frequent and substantially jeopardize clinical outcomes.^19,20^ Of note, the present study comprising over 1600 STEMI patients, followed for up to 10 years (median ∼4 years), observed that <20% of them developed CS or CA, both occurring in around 7% of them. This is aligned with the literature that shows similar incidence of such complications.^6–9^ It should be underlined that since the SHOCK trial performed many years ago, there have been very few randomized clinical trials in STEMI patients complicated with CS+, and very few registries on STEMI complicated with CA+ or CS+/CA+.^21^ Therefore, data for this challenging subset of patients are mostly derived from registries and single-centre analysis, with limited data on the long-term follow-up.

Several studies have evaluated the risk factors for mortality of CS and the use of different reperfusion strategies, antithrombotic therapy, and even the use of mechanical circulatory support (IABP, Impella, and ECMO).^22–24^ For instance, Garcia-Garcia *et al*. evaluated in-hospital and 1-year mortality of patients with STEMI complicated with CS from 1989 to 2018. In-hospital mortality in the 90 s was 61.3% and decreased significantly in the period 2014–18 to 43.7%.^6^ Therefore, in-hospital mortality has always been very high, and despite these initial improvements, there has been little reduction in the more recent decades.^25–28^ In our study, similar mortality rates were detected, with in-hospital mortality of 37% in the group with CS+, reaching 52% in the group with CS+/CA+. This highlights the dismal prognosis of the combination of both complications, especially among patients with a higher burden of comorbidities, such as age, chronic kidney disease, poorer ventricular function, and prior cardiovascular disease, well-known risk factors in such patients.^9,13^ Our study extends further such observations, with a much longer-term follow-up of almost 4 years. Of note, a three-fold higher mortality for CS+ or CA+ was observed, reaching approximately five-fold in the CS+CA+ group. Additionally, using flexible parametric models with B-splines, we estimated time-varying hazard and determined that the prognostic value of both CS+ and CA+ occurred within the first 10 months following STEMI, somewhat similar to prior studies that used landmark analysis at 1 year.^29,30^

Another important aspect is the question of whether the timing of these complications could play role in the prognosis.^17^ A prior study by Webb *et al*. has shown that 74.1% of the CS events occurred before 24 h (early shock) from the onset of STEMI symptoms with a median of 6.2 h. Of the patients with early shock, 62.6% died, while patients with later shock had 53.6% mortality.^31^ We also observed that the majority of CS and CA occurred in the first hours, before the patient underwent cardiac catheterization. Despite the differences in cut-off used for defining their timing of occurrence with respect to the initial event, our proportion of patients with pre-catheterization shock (38.5%) and during the procedure (31.9%) was very similar to the literature results for the first 24 h.^31^ This might be due to the fact CS+ patients presented more frequently with left descending artery involvement and concomitant multi-vessel disease, jeopardizing a larger burden of the left ventricle. Likewise, CS patients had greater times of symptoms onset to-hospital admission and symptoms onset to PPCI, highlighting the known importance of timely reperfusion in such patients.^20^ Finally, a quarter of patients developed CS during the hospitalization, and this is likely the result of infarction expansion, delayed inflammatory response, re-infarction, and concomitant coronary disease in other territories.^16,31^

Understanding the time of occurrence of CS can help in defining the best time to start the circulatory assist device. Of note, most studies such as IABP-Shock II and ESCL Shock have failed to show reduced mortality and outcomes with their routine use in CS patients.^28,32^ Most of these devices are inserted at the time of diagnostic cardiac catheterization, and this could already be a late stage. Danger Shock, a randomized study performed in only in three countries, was the first trial that showed a reduction in mortality with its routine use of Impella device in such patients, with an absolute risk reduction of close to 13% and a more upfront use.^33^ In addition, recent data have shown that even IABP use in earlier setting, according to the SCAI shock definition, might have a significant impact on the clinical outcomes.^34^

With regard to CA after STEMI, prior data refer to out-of-hospital events only, while in the present study, we included both in- and out-of-hospital events.^18^ This might explain the lower in-hospital mortality rates seen in our study of 20% vs. ∼41% of the literature, including such out-of-hospital CA patients. Also, with respect to the CS and CA timing of occurrence, CS impacted mortality at all timeframes, although CA only before and after cardiac catheterization impacted mortality, but not during the procedure.

We speculate that during PPCI, the occurrence of CA might be related to peri-procedural factors such as coronary instrumentation, anti-thrombotic therapy, distal embolization, reperfusion arrhythmias, and no reflow, among others, that are generally transitory and subject to immediate support, including cardiopulmonary resuscitation, defibrillation, and intracoronary medication administration.^11^ It is important to highlight the relatively small number of peri-procedural CA and wide CIs, so that we cannot draw a firm conclusion in this regard. CS and CA occurring after cardiac catheterization either alone or combined carried out the worst prognostic marker. After hospital discharge, guideline-oriented medication optimization has a critical role in improving clinical outcomes, although we recognize the small percentage of patients achieving such goals in the real world.^35^ In our study, this is underlined by the fact that p2y12 inhibitor and beta-blocker use at discharge were both associated with lower long-term mortality, although no specific method for medication compliance evaluation was used in the present study.

### Limitations

Although the present analysis comprises one of the largest cohorts of patients with STEMI presenting CS and CA, with very long-term follow-up, several limitations should be acknowledged. Given the large time frame of inclusion in the study practices, changes might have been incorporated, with possible impact on clinical outcomes. This is highlighted for instance by the high use of clopidogrel in the present study, rather than ticagrelor of prasugrel. In addition, we have no record of the use of ECMO in patients with CS, as well as the use of therapeutic hypothermia in patients with CA. Likewise, Swan–Ganz catheter was not systematically used in the present population; therefore, no haemodynamic data were available. Another limitation was that our data come from a single institution; however, our mortality rates and baseline characteristics are similar to other studies on CS. Also, out-of-hospital CS and CA were excluded from the present analysis, although the events at the referral centres or during transfer to our centre have been included. Lastly, despite all the statistical adjustments and sensitivity analyses, as any observational study, our observations should be regarded as associations, but no causal inferences can be done.

## Conclusion

In conclusion, CS and CA complicating patients presenting with STEMI have a significant association with higher long-term mortality rates, especially in the first 10 months. Both CS+ and CA+ at any timeframe impacted outcomes, except for CA+ during the initial cardiac catheterization, although this will have to be confirmed in larger future studies, given the relatively small number of patients.

## Supplementary Material

oeae075_Supplementary_Data

## Data Availability

The data underlying this article will be shared on reasonable request to the corresponding author.
