# Supplementary material for: Long-term outcomes of cardiogenic shock and cardiac arrest complicating ST-elevation myocardial infarction according to timing of occurrence
Source: Eur Heart J Open. 2024 Sep 3;4(5):oeae075. doi: 10.1093/ehjopen/oeae075 (PMC11430270; doi:10.1093/ehjopen/oeae075)
Supplement: oeae075_Supplementary_Data [file oeae075_supplementary_data.docx]

**Supplemental Table 1. Medication At Discharge.**

|  | **No cardiac arrest or shock** | **Cardiac arrest +** | **Cardiogenic shock +** | **Cardiac arrest + and cardiogenic shock +** | **p** |
| --- | --- | --- | --- | --- | --- |
| **N** | **1,263 (79.1%)** | **80 (4.9%)** | **138 (8.6%)** | **117 (7.2%)** |  |
| Clopidogrel | 972(77.0) | 67(83.8) | 90(65.2) | 78(66.7) | **0.001** |
| Aspirin | 1104(87.4) | 70(87.5) | 104(75.4) | 86(73.5) | **<0.001** |
| Beta Blocker | 1010(80.0) | 61(76.2) | 90(65.2) | 75(64.1) | **<0.001** |
| Statin | 1112(88.0) | 69(86.2) | 100(72.5) | 81(69.2) | **<0.001** |
| ACE Inhibitor | 961(76.1) | 64(80.0) | 88(63.8) | 66(56.4) | **<0.001** |

Values are n (%).

ACE Inhibitor = Angiotensin-Converting Enzyme Inhibitor

**Supplemental Table 2. In-Hospital Outcomes in 2 Periods of Time.**

|  | **2004-2009** | **2010-20217** | **p** |
| --- | --- | --- | --- |
| **N** | **581 (36.2%)** | **1022 (63.8%)** |  |
| Hospitalization (days) | 13 ± 16 | 12 ± 12 | **<0.001** |
| CCU (days) | 8 ± 10 | 9 ± 10 | 0.718 |
| LVEF (%) | 47 ± 12 | 46 ± 11 | 0.660 |
| IABP | 61 (12.8) | 101 (9.9) | 0.094 |
| In-hospital AMI recurrence | 51 (8.8) | 83(8.1) | 0.648 |
| Mechanical complications* | 33 (5.6) | 16 (1.5) | **0.034** |
| Stroke | 3 (0.6) | 12 (1.2) | 0.327 |
| Complete heart block | 30 (6.3) | 42 (4.1) | 0.064 |
| Temporary pacemaker | 45 (7.9) | 66 (6.5) | 0.287 |
| Permanent pacemaker | 1 (2.2) | 3 (4.6) | 0.510 |
| Atrial fibrillation/flutter | 45 (9.4) | 62 (6.1) | **0.019** |
| Sepsis | 81 (17.1) | 82 (8.0) | **<0.001** |
| Acute Renal Failure | 44 (9.3) | 132 (12.9) | **0.042** |
| Dialysis | 15 (4.0) | 32 (3.1) | 0.411 |
| Major bleeding | 21 (4.4) | 26 (2.5) | 0.052 |
| Blood transfusion | 0 (0) | 36(3.7) | 0.697* |
| Overall death | 133 (22.9) | 122 (11.9) | **<0.001** |
| Cardiovascular death | 56 (9.6) | 41 (4.0) | **<0.001** |
| Death due bleeding | 0 (0.0) | 4 (0.4) | 0.131* |
| Death due sepsis | 20 (3.4) | 21 (2.1) | 0.091 |

Values are n (%), mean ±SD or median [IQR].

CCU = critical care unit; LVEF = left ventricular ejection fraction; IAB = intra-Aortic balloon pump; sustained VT = sustained ventricular tachycardia; AMI = acute myocardial infarction; CV death = cardiovascular death.

*Mechanical complications included: cardiac tamponade, intraventricular communication, cardiac perforation, and mitral regurgitation.


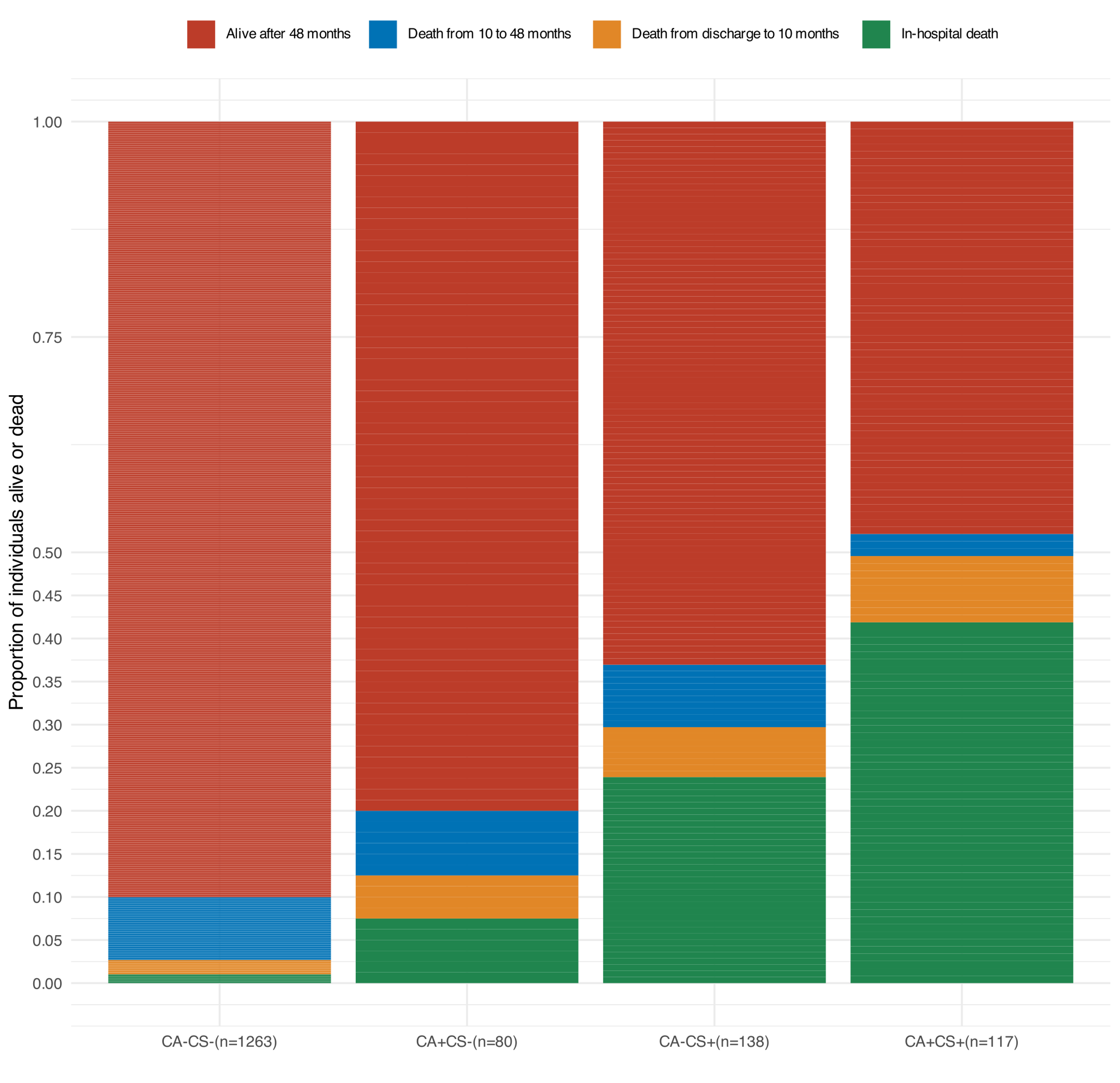


**Supplemental Figure S1.** Proportion of deaths occurring in-hospital, discharge to 10 months and 10-48 months.

**Supplemental Figure S2**. Model with 24 variables, including 10 "forced" variables in the model stepwise (Prior DM, Family History of CAD, BNP, Platelet – Lower, Glucose, Multivessel Disease, Medical Rx, CABG, Timi Flow = Zero, Hours of Symptoms-Reperfusion)

**Supplemental Figure 3. Timing of occurrence of both cardiac arrest (CA) and/or cardiogenic shock (CS) according to the cardiac catheterization.**

|  | CA+CS- | CA-CS+ | CA+CS+ |  |
| --- | --- | --- | --- | --- |
| n | 80 | 138 | 117 | p* |
| **Pre-Cath CS (%)** | - | 52(38.5) | 65(58.6) | 0.083 |
| **During-Cath CS (%)** | - | 43(31.9) | 29(26.1) | 0.40 |
| **Post-Cath CS (%)** | - | 40(29.6) | 17(15.3) | 0.026 |
| **Pre-Cath CA (%)** | 46(59.0) | - | 66(58.9) | 0.93 |
| **During-Cath CA (%)** | 15(19.2) | - | 17(15.2) | 0.50 |
| **Post-Cath CA (%)** | 17(21.8) | - | 29(25.9) | 0.64 |
| **Schockble Rhythm (%)** | 73(91.2) | - | 86(73.5) | 0.31 |

* Bonferroni post-hoc p-values

**Supplemental Figure S4.** Stepwise Cox regression model for all-cause long-term mortality, including a forced variable door-to-balloon


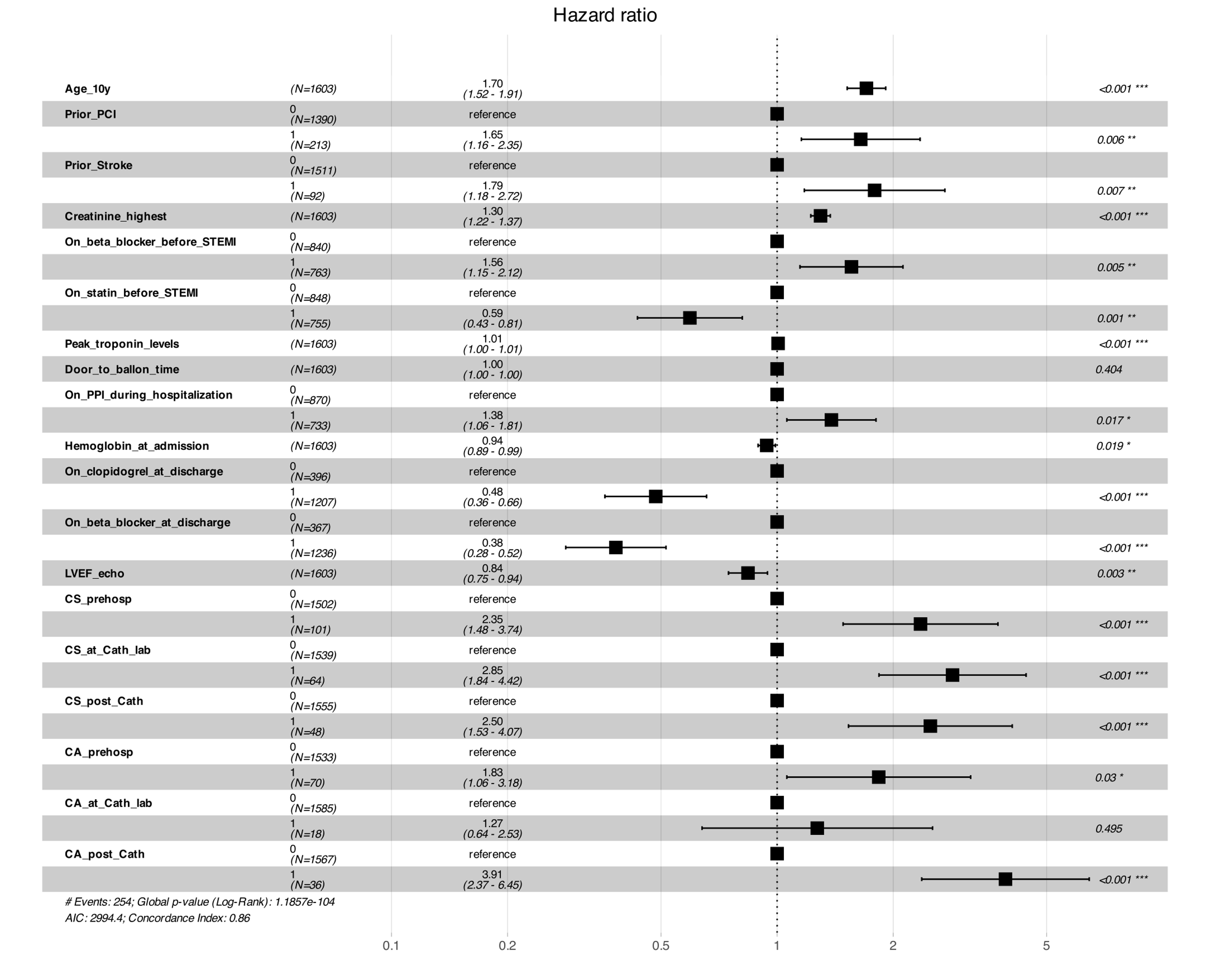


**Door-to-Balloon Time**

**Age (10 Years)**

**Prior PCI**

**Prior Stroke**

**Creatinine Peak**

**On Beta Block before STEMI**

**On Statin before STEMI**

**Troponin Peak**

**On PPI During Hospitalization**

**Hemoglobin at Admission**

**On Clopidogrel at Discharge**

**On Beta Block at Discharge**

**LVEF (Echo)**

**Pre-Cath CS**

**During-Cath CS**

**Post-Cath CS**

**Pre-Cath CA**

**During-Cath CA**

**Post-Cath CA**
